# Current trends in graft choice for anterior cruciate ligament reconstruction – part I: anatomy, biomechanics, graft incorporation and fixation

**DOI:** 10.1186/s40634-023-00600-4

**Published:** 2023-04-01

**Authors:** Armin Runer, Laura Keeling, Nyaluma Wagala, Hans Nugraha, Emre Anil Özbek, Jonathan D. Hughes, Volker Musahl

**Affiliations:** 1 Department of Orthopaedic Surgery, UPMC Freddie Fu Sports Medicine Center, University of Pittsburgh, Pittsburgh, PA USA; 2 Department for Sports Orthopaedics, Klinikum Rechts der Isar, Technical University of Munich, Munich, Germany; 3 Department of Orthopaedic and Traumatology, Faculty of Medicine, University of Udayana, / Prof. Dr. I.G.N.G. Ngoerah General Hospital, Denpasar, Bali Indonesia; 4 Department of Orthopedics and Traumatology, Ankara University, Ankara, Turkey; 5 Department of Orthopaedics, Institute of Clinical Sciences, Sahlgrenska Academy, University of Gothenburg, Gothenburg, Sweden

## Abstract

Graft selection in anterior cruciate ligament (ACL) reconstruction is critical, as it remains one of the most easily adjustable factors affecting graft rupture and reoperation rates. Commonly used autografts, including hamstring tendon, quadriceps tendon and bone-patellar-tendon-bone, are reported to be biomechanically equivalent or superior compared to the native ACL. Despite this, such grafts are unable to perfectly replicate the complex anatomical and histological characteristics of the native ACL. While there remains inconclusive evidence as to the superiority of one autograft in terms of graft incorporation and maturity, allografts appear to demonstrate slower incorporation and maturity compared to autografts. Graft fixation also affects graft properties and subsequent outcomes, with each technique having unique advantages and disadvantages that should be carefully considered during graft selection.

## Introduction

The primary goal of ACL-R is restoring antero-posterior and rotatory knee stability and function as closely as possible to the native joint. Despite advances in surgical techniques and rehabilitation, postoperative complications including graft rupture remain significant, yielding severe socioeconomic consequences and detrimental patient experience.

Revision surgery rates average between 2 and 10% [32, 39, 90, 91, 98, 128] but may be as high as 42% in high-level pivoting athletes [27, 29, 62, 96, 97]. Several well-known intrinsic and extrinsic risk factors, including patient age, activity level, and alignment influence postoperative outcomes and failure rates [54, 81, 96, 128]. Graft choice has been highlighted as an adjustable extrinsic factor with impact on failure of ACL-R [54, 96, 98].

Graft choices in ACL-R are broadly divided into autograft and allograft tissue. Hamstring tendon autograft (HT) is the most commonly used autograft among ACL surgeons worldwide, followed by bone-patellar-tendon-bone (BPTB) and quadriceps tendon autograft (QT) [7]. When available, allograft presents an attractive alternative to autograft due to shorter surgical time and avoidance of donor site morbidity. Numerous allograft sources are available, including all-soft tissue as well as tendon-bone options.

The following review aims to highlight current concepts of graft choice in ACL-R and provide the most up-to-date evidence regarding the graft selection process for primary ACL-R. The first of two parts, this paper will discuss the anatomical, biomechanical, and histological properties as well as differences in graft incorporation and fixation techniques of the three most widely used autografts and allografts. The second part will focus on clinical outcomes, failure rates and complications associated with each graft option.

### Graft choice rationale

Individualized graft choice is advised in modern ACL-R; no single graft is appropriate for all patients. When choosing the optimal graft for each patient, the surgeon must consider multiple patient-specific, physician-specific, and graft-specific factors. Such considerations include tissue availability, prior or concomitant injury, patient comorbidities, and surgeon experience. The optimal graft will offer an expeditious harvest with low morbidity, rapid graft integration, and mechanical and structural properties similar to the native ACL. Despite this, each graft option has unique anatomical and biomechanical characteristics with resultant advantages and disadvantages.

## Anatomy and microstructural properties

Successful ACL-R necessitates reconstruction of native anatomy. A profound comprehension of ligamentous anatomy is the first step in the graft selection process.

### Native ACL

ACL-R is predominantly performed as a single-bundle procedure. Quantitative measurements of the native ACL are patient-dependent with length, cross-sectional area (CSA), and volume ranging from 26 to 38 mm [2, 25, 36, 42, 118], 30 to 53 mm^2^ [17, 25, 36, 109, 110, 119, 124] and 854 to 1858 mm^3^ [66, 122, 123], respectively. Descriptions of the femoral origin and tibial insertion sites vary in CSA and morphology The femoral CSA ranges between 60 and 130 mm^2^, whereas a larger CSA (from 100 to 160 mm^2^) has been described for the tibial site [36, 55–58, 67, 68, 85, 107, 108, 114, 117].

Histologically, the native ACL demonstrates a high percentage of fibroblasts, blood vessels, and elastic fibrils, with a relatively low ratio of collagen fibrils to interstitium. These characteristics facilitate ACL function during daily activity, as they allow for regeneration and enable the ligament to withstand multiaxial stresses and fluctuating tensile strains [46].

### Autograft

There are several different autograft options available for ACL-R, the most prevalent of which include BPTB, QT and HS. In general, each graft should be at least 7 cm long and have a midsubstance CSA similar to the native ACL.

The BPTB autograft represented historically the “gold standard” in ACL-R. The graft consists of an approximately 10 mm wide tendon strip obtained from the central third of the patellar tendon and includes two bone blocks, one each from the tibial tuberosity and the patella. Compared to HT it is more “flat” and has less collagen fibers compared to QT [45].

Unlike the BPTB autograft, multiple configurations are described for the QT autograft. It can be harvested with or without a bone block and as an approximately 10 mm wide full-thickness graft, or a 12 × 5 mm partial-thickness graft [34]. Histologically, the QT provides approximately 20% more collagen fibrils and a higher density of fibroblasts than a BPTB autograft of the same size, with comparable thickness of collagen fibrils and density of blood vessels [45]. Although some have cited concerns regarding mismatch between patient height and QT graft size, the literature demonstrates that QT autograft of sufficient length and thickness can be obtained in patients with small stature [40].

For HT autograft, harvested from the semitendinosus and/or gracilis tendon, there is wide variability in graft configurations ranging from one to eight strands, with quadrupled hamstring being the most common [75]. While BPTB and QT autograft are generally consistent in terms of length and thickness, hamstring tendons are correlated with patients’ anthropometrics and sports activity level and are therefore patient-dependent [89, 121]. Graft size does not correlate with ACL footprint size [57]. Microscopic analysis of HT autograft demonstrates a 20% to 40% higher number of collagen fibrils and fibroblasts compared to patellar tendon autografts [47].

When comparing the CSA of the BPTB (33 – 61 mm^2^) [50, 57, 85, 105], HT (52 – 64 mm^2^) [50, 57, 85], and QT (71 – 91 mm^2^) [50, 85, 105] autografts to the intact ACL, the QT appears to most closely approximate the size of the native footprint. These descriptive data are supported by a cadaveric study comparing the microscopic anatomy of BPTB and QT autograft, showing more favorable femoral insertion width, insertion thickness, and graft bending angle for the QT autograft [64].

When comparing histological features of commonly used autografts, none can replace the complex ultrastructural characteristics of the native ACL [16, 46]. The native ACL has a lower collagen fibril to interstitium ratio, yet higher fibroblast, elastic fibril, and blood vessel density compared to all autograft options [46]. A high percentage of collagen fibrils in tendon and ligament is associated with increased structural properties, but negatively influences elasticity and tendon constriction [46].

### Allograft

Allografts can be generally subdivided into all-soft tissue and bone-tendon grafts. Soft tissue allografts include hamstring, tibialis anterior, tibialis posterior, peroneal tendon, and iliotibial band/fascia lata, while subtypes of bone-tendon allografts are BPTB, QT with patellar bone block, or Achilles tendon with calcaneal bone block. Similar to autograft options, BPTB allograft is the only allograft with bone blocks on either tendon side, and therefore the only option providing femoral and tibial bone-to-bone healing. While allografts have similar anatomical properties to their autograft correlates, the use of allograft offers the option of customizing graft size to the individual patient’s anatomy.

## Biomechanics

When considering biomechanical studies of the native ACL and its respective graft options, it is important to recognize that numerous factors influence outcomes, including experimental testing variables (temperature, storage, freezing and thawing time, specimen orientation, measurement techniques, loading rate), as well as patient or cadaver-specific factors (age, body weight, immobilization, or activities performed during the life of the donor) [126]. It is therefore inherent to biomechanical research that the results of individual studies vary greatly. It is also important to understand that biomechanical graft characteristics change during the healing process and therefore reflect only time zero. The following will review the biomechanical characteristics of the ACL in relation to various graft options, bearing in mind these limitations of biomechanical research.

### Ultimate load to failure

#### Native ACL

The primary and secondary functions of the ACL are to prevent anterior translation and internal rotation of the tibia, respectively, in relation to the femur. Studies on structural properties of the native ACL report an age- and sex-dependent ultimate load to failure of 2160 ± 157 Newtons (N) in young adults [127]. These values decrease over time to 658 ± 129 N in specimens older than 60 years of age [18, 127].

#### Autograft

The ultimate load to failure of BPTB autograft ranges from 319 to 4389 N, with the highest load reported in 15 mm-wide grafts [75]. In clinical practice, 10 mm-wide grafts with ultimate loads to failure of 1880 to 2664 N are typically used [26, 50, 111].

Similarly, the ultimate load to failure for a 10 to 12 mm-wide QT autograft ranges from 249 to 2186 N [50, 75, 111]. QT autograft with bone block, as well as full-thickness grafts appear to have higher ultimate loads to failure compared to all-soft tissue or partial thickness grafts [111].

For HT autograft, graft configuration (including total number of strands) correlates with graft size, which is in turn positively correlated with tensile strength [14]. Depending on graft configuration, graft diameters ranging from 6 mm to over 10 mm can be obtained with ultimate loads to failure ranging from 225 to 4590 N [50, 75, 111]. While a graft should have a minimum thickness of 8 mm, increased graft CSA is associated with an increased complication risk due to notch and PCL impingement [49, 74, 76, 89].

In a recent study by Hart et al. comparing the biomechanical properties of the three most common autografts, no statistically significant difference was found in ultimate load to failure among the graft options [50]. Thus, in terms of ultimate load to failure, all graft options appear to be viable substitutes for the native ACL.

### Stiffness

To restore normal knee kinematics and physiologic joint forces the stiffness of the used graft should be similar to the native ACL. Supraphysiologic graft stiffness results in knee over-constraint and increased chondral stress, thereby increasing the risk of early onset osteoarthritis [48, 112].

#### Native ACL

Values for native ACL stiffness are reported to be 242 ± 28 N/mm in young adults. As with ultimate load to failure, these values decrease with age to 180 ± 25 N/mm in patients over 60 [127].

#### Autograft

For BPTB grafts, stiffness is reported to range from 158 to 685.2 N/mm, with values between 324 and 543 N/mm for grafts of 10 mm width [3, 75, 111]. For QT, stiffness is reported to be between 17.0 and 809.0 N/mm, with the smallest values seen by Noyes et al. when testing a quadriceps tendon-patellar retinaculum-patellar tendon graft construct [83]. A similarly wide range of stiffness (4.1 to 1148.0 N/mm) has been reported for HT autografts due to the variability in graft configurations [75].

When comparing all three graft options, Hart et al. [50] found a significantly higher stiffness for QT (672 ± 210 N/mm) compared to four-stand HT (397 ± 91 N/mm), yet similar values when compared to BTPB (543 ± 73 N/mm). In contrast, Strauss et al. [111] reported higher cyclic loading stiffness values for HT (273 ± 49.5 N/mm) compared to BPTB (151 ± 25.5 N/mm) and QT (157 to 173 N/mm, depending on configuration).

In summary, graft stiffness is an important factor in graft choice for ACL-R. At time zero, none of the grafts can perfectly mimic the native ACL and little evidence exists thereafter. It seems that the HT graft has the highest tendency towards supraphysiologic stiffness.

### Modulus, stress and strain

#### Native ACL

Modulus of elasticity for the native ACL is reported to be between 111 and 124 MPa [18, 84]. This is generally lower than the reported moduli for ACL graft options; a recent systematic review including 26 biomechanical studies of commonly used grafts reported higher ranges for each of the three most prevalent autograft options, as well the majority of allografts [75].

#### Autograft

Modulus, maximum stress, and failure strain for BPTB range from 184 to 337.8 MPa, 21.6 to 101.3 MPa, and 0.16 to 25%, respectively. For QT, the same values range from 153.0 to 255.3 MPa, 9.7 to 23.9 MPa, and 2.0 to 10.7%. HT values are reported to be as high as 144.8 to 904.0 MPa, 65.6 to 156.0 MPa, and 0.3 to 33.0%, respectively [92].

#### Allograft

As with autografts, the structural and mechanical characteristics of allografts differ depending on harvest site. Common allograft options frequently meet or exceed the biomechanical properties of the native ACL [65]. For single-stranded grafts, the lowest and highest load to failure are reported for tibialis anterior and quadriceps tendon allografts, respectively [5, 65, 105]. While gender does not appear to have an effect on allograft properties [61], older donor age has been negatively correlated with biomechanical characteristics [13, 41, 61, 116].

### Allograft processing

In addition to donor characteristics, graft preservation techniques alter the properties of allograft tendon. These changes are important to recognize when considering the use of allograft. Gamma irradiation and electron beam (E-beam) are employed for inactivation of bacteria and other pathogens. Mixed effects have been reported for low-dose gamma irradiation (< 20 kGy), with little [28, 130] or no decrease in stiffness and ultimate load to failure [11, 41, 78]. However, a positive dose-dependent effect of high irradiation is seen on mechanical tendon properties, altering the integrity of the tendon with a decrease in ultimate load to failure of up to 74% compared to non-irradiated tissue [9, 33, 38, 78, 104]. Similarly, E-beam irradiation produces detrimental effects on structural properties [43, 52], albeit to a lesser extent than gamma irradiation [51]. Varied biomechanical effects have also been reported for chemical sterilization including peracetic acid, BioCleanse1 (RTI Surgical, Inc), ethylene oxide, or supercritical CO2 treatment [5, 8, 30, 61, 100, 101, 103].

Methods of preservation also influence tendon properties [37, 113]. Freezing a tendon at -80 °C increases the mean diameter of collagen fibrils, while the mean number of fibrils decreases. Biomechanically, this corresponds to a decrease in ultimate load (decrease of 82% compared to fresh frozen), ultimate stress (decrease of 70% compared to fresh frozen), and ultimate strain, yet an increase in stiffness [37]. Furthermore, multiple freeze–thaw cycles appear to affect histological and biomechanical tendon properties, although study results remain contradictory [19, 63, 115]. Alternative preservation techniques like glycerolization, lyophilization, or preservation with chloroform–methanol extraction may also lead to a 50% decrease in the structural and mechanical properties of the allograft [43, 133].

In summary, fresh frozen allograft tissue may meet or exceed the biomechanical characteristics of the native ACL, however various sterilization and preservation methods alter histological and biomechanical graft properties. While low dose irradiation appears to have little influence on graft biomechanics, moderate- to high-dose irradiation and chemical processing have detrimental tissue effects and should be avoided when possible.

## Graft incorporation

Much of our current knowledge about graft incorporation derives from animal studies. It should be noted that animal studies carry potential bias, including time-dependent differences in soft tissue remodeling compared to humans. Furthermore, postoperative immobilization and physiotherapy, both recognized in optimizing graft incorporation, cannot often be performed in animals. Therefore, these studies should be used cautiously when treating and advising patients [65].

Graft remodeling occurs within the first six months postoperatively and may continue for years [1, 22, 71, 125, 131]. During this time, the implanted tendon undergoes a remodeling where the composition and organization of the tendon are adapted to new intraarticular conditions and functions [102]. When compared to BPTB autograft, HT autograft appears to have delayed progression (6 to 12 months vs. 12 to 24 months) of remodeling [1, 31, 60, 95, 99]. Similarly, in one study superior graft maturity was observed for QT autograft with bone block versus HT autograft at six months postoperatively [73], although a second study reported no difference [87]. The results of earlier studies of graft maturation have been recently challenged using quantitative MRI UTE-T2* and T2* mapping, showing no difference in maturation between BPTB and HT autograft [22]. Furthermore, graft maturation has not been correlated with clinical outcome and rotatory knee stability one and two years after HT ACL-R [69, 71].

Graft-to-bone integration is necessary for optimal healing and resemblance of the physiologic ACL [88]. Early histological and biomechanical animal studies suggest that bone-to-bone healing is faster and stronger compared to tendon-to-bone healing (8 vs. 12 weeks) [6, 73, 88, 93, 120]. However, this widely accepted theory has been disputed by a recent in vivo human study showing similar graft-tunnel motion at 6 and 12 months postoperatively between BPTB and HT autograft, suggesting that bone-to-bone may not be necessarily faster than ligament-to-bone healing [59].

Animal studies also suggest that higher graft-to-bone contact area has positive effects on tendon–bone healing, especially in the early period after ACL-R [12, 23, 132]. Additionally, healing is sensitive to dynamic changes in graft forces, with early high forces on the ACL graft appearing to impair graft-tunnel osseointegration [72].

## Graft fixation

With the advent of faster and more aggressive rehabilitation protocols, the primary aim of graft fixation is to provide stability of the graft within the bone tunnel until graft-to-bone incorporation is accomplished. Optimal graft fixation minimizes graft elongation, longitudinal (“bungee effect”) and transverse (“windshield wiper”) graft movement, as well as influx of synovial fluid into the bone tunnel by maximizing strength, stiffness, stability, and durability. Despite advancements in graft fixation methods, the fixation point remains the weakest link in the graft-to-bone interface and is therefore crucial to the success of ACL-R.

Several direct and indirect methods of graft fixation have been described. Direct methods include absorbable and non-absorbable interference screws, cross pins, staples, washers, or hardware-free press-fit fixation, whereas indirect devices include fixed or adjustable suspensory cortical button fixation. At this point, there is no clear consensus regarding the “best” graft fixation method, as each option has advantages and disadvantages. Several recent meta-analyses [20, 24, 53, 82, 106] and network meta-analyses [53, 129] have demonstrated no superiority in clinical or patient-reported outcomes (PROs) of any particular fixation method. However, a recent meta-analysis of 40 studies found improved arthrometric stability and fewer graft ruptures but no difference in PROs using suspensory- compared to interference screw fixation for quadrupled HT autograft [15].

Advantages of suspensory fixation include the ease and simplicity of technique, the possibility of a thicker graft with higher graft-to-bone contact area resulting in superior graft incorporation, as well as excellent fixation strength and stiffness [23, 35, 77, 79]. When comparing fixed loop- to adjustable loop suspension, superior biomechanical results have been observed for fixed loop devices [86, 92]. Compared to interference screws, less tunnel widening is seen when using suspensory fixation or cross pins, which becomes relevant in revision cases [21, 35, 80]. Graft elongation as well as longitudinal and transverse movements appear to be lower using interference screws, especially when screws are placed close to the joint surface [70, 77, 94].

Hardware-free press-fit techniques have been reported, showing promising outcomes comparable to traditional techniques with low rates of tunnel enlargement [4, 10, 44, 106].

## Conclusion

Graft choice has a considerable influence on postoperative outcomes and remains an easily adjustable surgical factor affecting graft rupture and reoperation rates. When comparing anatomical, histological, and morphological features of commonly used grafts to the native ACL, none can perfectly replicate the complex characteristics of the native ACL. Biomechanically, however, both autograft and allograft show equivalent or increased characteristics compared to the native ACL and represent viable options for ACL-R. There further remains limited evidence as to the superiority of one graft in terms of maturation and incorporation, yet the available literature suggests that allograft may demonstrate slower graft incorporation and maturity compared to autograft tissue. Finally, methods of graft fixation have unique advantages and disadvantages that affect graft properties, and should be carefully considered when selecting the optimal graft for each patient.

**Table:** 

		**Advantages**	**Weaknesses**
**Anatomy**	**QT**	QT up to 20% more collagen fibers and a higher density of fibroblasts than BPTB Possibility of different harvest configurations Largest CSA	Sometimes short graft
	**BPTB**	Possibility to harvest with bone block on each site	Smallest CSA of all grafts Not able to replace the complex ultrastructural characteristics of the native ACL
	**HT**	Possibility of different graft configurations to individualize graft thickness	Unpredictable tendon thickness
	**Allograft**	All possible graft configurations depending on the used tendon Customizing graft size to the individual patient’s anatomy	Processed tissue
**Biomechanics**	**QT**	Similar load to failure than BPTB but higher than native ACL	Two layers may sometimes separate
	**BPTB**	Similar load to failure than QT but higher than native ACL	Bone tendon junction may have tendinosis
	**HT**	Common graft configurations exceed the load to failure of the native ACL	Load to failure depending on graft configuration Tendency to supraphysiologic stiffness if multistrand graft
	**Allograft**	Highest load to failures reported for the quadriceps tendon allograft	Older donor age negatively correlated with biomechanical characteristics Graft sterilization and preservation techniques influence biomechanical graft properties
**Graft Incorporation**	**QT**	Faster incorporation compared to HT autograft Possibility for one-sided bone-to-bone healing	Short tendon-tunnel interface
	**BPTB**	Faster incorporation compared to HT autograft Possible faster graft incorporation due to bone–to–bone healing	Size mismatch
	**HT**		Delayed incorporation compared to BPTB and QT no possibility of bone-to-bone healing
	**Allograft**		Slower graft maturation process as well as slower onset and rate of revascularization
